# Trajectories of aggressive and depressive symptoms in male and female overweight children: Do they share a common path or do they follow different routes?

**DOI:** 10.1371/journal.pone.0190731

**Published:** 2018-01-05

**Authors:** Luca Cerniglia, Silvia Cimino, Michela Erriu, Stanislav Jezek, Carlos A. Almenara, Renata Tambelli

**Affiliations:** 1 International Telematic University Uninettuno, Psychology Faculty, Department of Psychology, Rome, Italy; 2 Sapienza, University of Rome, Psychology and Medicine Faculty, Department of Dynamic and Clinical Psychology, Rome, Italy; 3 Institute for Research on Children, Youth and Family, Faculty of Social Studies, Masaryk University, Brno, Czech Republic; 4 Universidad Peruana de Ciencias Aplicadas, Faculty of Psychology, Chorrillos, Lima, Perú; TNO, NETHERLANDS

## Abstract

The prevalence of childhood overweight is a major social and public health issue, and primary assessment should focus on early and middle childhood, because weight gain in these phases constitutes a strong predictor of subsequent negative outcomes. Studies on community samples have shown that growth curves may follow linear or non-linear trajectories from early to middle childhood, and can differ based on sex. Overweight children may exhibit a combination of physiological and psychosocial issues, and several studies have demonstrated an association between overweight and internalizing/externalizing behavior. Nevertheless, there is a dearth of longitudinal studies on depressive and aggressive symptoms in children with high BMI. This study adopted a growth curve modeling over three phases to: (1) describe BMI trajectories in two groups of children aged 2–8 (overweight and normal weight) from a community sample; (2) describe the developmental trajectories of children’s aggressive and depressive symptoms from 2 to 8 years of age. Results indicate higher BMI in 2-year-old girls, with males catching up with them by age 8. While overweight females’ BMIs were consistently high, males’ increased at 5 and 8 years. The mean scores for aggressive symptoms at T1 (2 years of age) were the same in all subjects, but a significant deviation occurred from T1 to T2 in both samples, in divergent directions. With regards to children’s depressive symptoms, the two groups had different starting points, with normal weight children scoring lower than overweight youths. Overweight females showed lower depressive scores than overweight males at T1, but they surpassed boys before T2, and showed more maladaptive symptoms at T3. This study solicits professionals working in pediatric settings to consider overweight children’s psychopathological risk, and to be aware that even when children’s BMI does not increase from 2 to 8 years, their psychopathological symptoms may grow in intensity.

## Background

Several studies have demonstrated that the prevalence of overweight (OW) in children has significantly increased over the last two decades [1]. Overweight in childhood has been found to predict severe negative health outcomes in adolescence and adulthood, such as cardiovascular diseases, Type II diabetes, asthma, obstructive sleep apnea, and hyperlipidemia [2]. The increasing prevalence of childhood overweight constitutes such a major social and public health issue that the World Health Organization has strongly recommended applying growth-monitoring policies to all children aged under 17 to inform prevention and intervention strategies [3]. Moreover, it has been posited that primary assessment and intervention should focus on children in early to middle childhood, because weight gain in this phase constitutes a strong predictor of adiposity in later life [4]. In the present paper, we use the definition of overweight developed by the International Obesity Task Force (IOTF), which proposed an international Body Mass Index (BMI, kg/m2) reference standard to define underweight, overweight, and obesity according to children’s age, weight, and height [5,6]. Furthermore, we also consider overweight children whose mothers experienced no peri- or post-partum difficulties or health problems, who were not born preterm, and who were of average socioeconomic status [7].

It has been suggested that BMI of children in the first years of life may follow different developmental trajectories in the general population, particularly from 3 years of age [8]. Studies examining these growth trajectories have identified diverse patterns of change in weight status during infancy and childhood [9,10,11]. These studies suggest that growth curves may follow linear or non-linear trajectories, with children belonging to overweight groups maintaining a high BMI over time, or else linearly decreasing or increasing from 2 to 10 years of age. In other cases, overweight children’s BMI has been shown to decrease from early to middle childhood and then “rebound,” increasing again at 6–8 years [12]. Several studies have focused on how these changes in BMI during growth are attributable to genetic or early life factors [13,8,14], as well as how these changes can predict obesity later in life [15]. It is important to note that these changes in growth are accompanied by physiological and psychological changes that can have interactive effects and most likely play an important role in the establishment and development of psychopathology [16].

However, the developmental relationship between weight status and mental health is very complex [17]. Overweight children frequently show a combination of physiological and psychosocial issues, such as abnormal development of interoceptive awareness (i.e., deficits in recognizing hunger and satiety cues [18]), peer problems, mood disorders, and low self-esteem, among other issues that undermine their quality of life [19,20,21,22,23]. They also tend to suffer from social stigma connected to their weight, which can have negative psychosocial outcomes, especially in late childhood and adolescence [24,25]. According to Vila et al. [26], more than 60% of overweight children suffer from some form of psychological difficulties, but this figure suggests that there is still a substantial proportion of overweight children who do not face psychological problems. In line with this, the “Jolly Fat” hypothesis proposes that some obese individuals display better mental health than otherwise similar normal weight individuals [27]. However, the impact of being overweight, particularly in the case of emotional difficulties, seems to be due to the extent of the negative impact of this condition on children’s self-concept [26,28]. During early childhood, the self-concept is characterized by inaccurate self-appraisals mostly due to the immature cognitive development at this early age [29]. For instance, early age children lack the ability to engage in social comparisons to conclude if they are more or less competent than their peers [30]. But later, preschool children become much more aware of the negative attitudes and prejudices toward obese individuals and start to engage in social comparisons more frequently. Thus, the developmental interplay of self-appraisals and social comparisons [31] could explain the variation in how being overweight affects children’s self-esteem and in turn their mental health and quality of life. Indeed, the association between obesity and lower self-esteem seems much more established in later ages such as adolescence (e.g., [1]). Similarly, developmental interplay with other factors such as gender or cultural norms can influence the association between obesity and poorer mental health [2,3].

Looking at the development of behavioral and emotional problems, Camfferman et al. [32] have demonstrated an association between overweight and internalizing/externalizing behavior in early childhood based on several different mechanisms. *Externalizing* behavior and high BMI in children may share individual risk factors such as impaired self-regulation skills and difficult temperament, but children’s overweight can be also associated with parent-child relationship issues, e.g. inadequate parental behavior and feeding practices. For instance, parents could respond to a child with a difficult temperament by proposing sedentary activities (e.g., watching television), or they could use food as a positive or negative reinforcement to calm down the child, which may eventually result in overweight [33]. Similarly, *Internalizing* problems may share individual risk factors with high BMI, such as dysregulated serotonin and cortisol levels, which can be associated with overweight as a consequence of weight stigmatization, rejection by peers, and frustration about the impossibility of participating in certain physical activities due to excessive weight [34]. It has also been shown that children may use food as a means to control and dump negative emotions (e.g., depressive symptoms), and that parents may offer food to comfort their children and reduce their anxiety and stress [35]. Children may therefore find themselves locked in a maladaptive circular process, where internalizing symptoms foster overweight, which in turn leads to more internalizing problems, and so forth. Although an association between overweight and internalizing/externalizing problems has been widely demonstrated, few studies have focused on this association in early childhood and from a longitudinal perspective [36]. The use of a prospective standpoint is crucial, as Sawyer et al. [37] posited, in that the association between overweight children’s BMI and psychological problems may only emerge in middle childhood, meaning that this correlation could not be present in earlier years of life. Moreover, few studies have addressed specific psychopathological symptoms in overweight children, as most authors considered overall internalizing and externalizing problems [38]. A limited (but growing) body of literature has investigated the association between overweight in children and specific “core” aspects of internalizing and externalizing problems in longitudinal studies (such as, for example, depressive symptoms). However, these studies mostly addressed the predictive power of children’s [39,40] or adolescents’ [41] depression (and psychopathology in general) on the risk of obesity in adolescence and adulthood [42], rather than focusing on trajectories of psychopathological profiles and their associations with overweight *within* the childhood developmental stages (early childhood; middle childhood). Several authors have addressed depression in children and its associations with overweight in longitudinal studies, but they mostly concentrated on samples of youths from 6 to 18 years of age [43,44]. Some scholars have indeed studied trajectories of overweight and psychopathological symptoms from early childhood to adolescence, but their research included an assessment of depression only in adolescence, whereas other measures (e.g., children’s BMI) were administered from the start of the study [45,46].

Importantly, most studies on BMI trajectories have examined boys and girls together, whereas fewer authors have focused on different trajectories in males and females in the first years of life [47]. The few exceptions have revealed gender differences in BMI trajectory patterns [48]. Girls, for example, may be more significantly affected by factors associated with biological variations in the onset of puberty and by the strong relationship between sexual maturation and changes in adiposity. Moreover, it has been demonstrated that sex plays a role in predicting the association of psychopathological symptoms and high BMI. Externalizing symptoms have been predominantly found in overweight males, whereas internalizing problems are more prevalent in females [49]. The fact that literature in the field has widely accepted that aggressive behaviors are more prevalent in boys than girls means that there is a dearth of recent studies on conduct problems in overweight females [50], impeding the development of informed prevention and intervention programs. A gap in the literature likewise exists concerning middle childhood and the paths BMI may follow in that specific developmental stage [51]. Due to mental health concerns, most research linking children’s BMI and psychopathological risk has been conducted on clinical samples [52]. Nevertheless, it has been suggested that assessing maladaptive emotional and behavioral patterns in children from community samples could better inform prevention and intervention policies [53].

In this paper we adopted a growth curve model with two objectives: (1) to describe BMI trajectories from age 2 to 8 years in two groups of children (overweight and normal weight) of both sexes from a community sample; (2) to describe the developmental trajectories of children’s aggressive and depressive symptoms from 2 to 8 years.

## Methods

### Sample and procedure

In 2006, our research group started a screening program in collaboration with pediatricians working in public and private kindergartens and schools in Central Italy. The aim was to detect overweight children and to examine their trajectories over time in terms of both BMI and emotional-behavioral functioning. The study protocol was approved by the Ethical Committee of the Psychology Faculty of Sapienza—University of Rome, in accordance with the guidelines approved by the Helsinki Declaration. Additionally, participating schools obtained ethical clearance through their respective institutional review bodies. The study was conducted in three phases each three years from ages 2 to 8 (T1 to T3). Our collaborating group of pediatricians and psychologists carried out the physical and psychological assessments (see Tools section below). At T1, in 2006, over a period of 6 months, we recruited 90 children (data are available in Mendeley Datasets at 10.17632/93cdm7mc3d.1). The sample of overweight children was balanced by sex and randomly selected using computer software from among all the two-year-old children above the 85th percentile for weight included in the screening program (Overweight group/OWg). The assessment was made by pediatricians at kindergartens and schools using the World Health Organization (WHO) growth curves [6], in the absence of any referred medical and/or psychiatric diagnosis. OWg was paired with a sample of N = 90 children (randomly selected from among the same school population) in which 2-year-old children presented with adequate weight and without further medical and/or psychiatric impairment (Normal Weight group/NWg). Informed written consent was obtained from all children’s parents for the aims of the study, and at each phase of the study, both parents completed a scale measuring their child’s emotional-behavioral functioning (see Tools section below). This procedure was repeated for each phase, and the sample was not affected by attrition thanks to the collaboration of pediatricians and schools, which strongly supported the program. The team of pediatricians and psychologists were the same for every assessment point. Screening results were sent to parents after each measurement and contained the child’s BMI-for-age percentile and a description of the outcomes and suggested actions, if any.

### Tools

#### Body Mass Index (BMI)

BMI is a composite measure of children’ weight and height considering age. To identify overweight children, we used international cut-off points [6].

#### Children’s emotional/behavioral problems

We used the two Italian versions of the Child Behavior Checklist (CBCL) [54]. One is designed for 1½–5-year-old children and the other one is for 6–18-year-olds [55]. Both versions contain items assessing internalizing/externalizing problems, although the formulation of some items differs across versions to be coherent with the developmental changes within these domains. As this paper uses a growth curve model, we needed measurement equivalence across time points [56]. Thus, consistent with the study of Sterb et al. [57], we chose items from both versions of the CBCL known to have greater developmental invariance [58]. Moreover, we identified “core” aspects of internalizing and externalizing syndromes that are developmentally appropriate and phenotypically expressed from preschool to pre-puberty [59]. Consequently, we created subsets named “Depression” and “Aggression.” The Depression subset was composed of items 47, 68, 70, 87, 98 in the version of the test for 1½–5-year-olds and 5, 50, 65, 70, 71 111 in the version for 6–18-year-olds. The Aggression subset was composed of 18, 20, 40, 53, 66, 81, 85, 96 in the version of the test for 1½–5-year-olds and 19, 21, 22, 37, 57, 68, 86, 95 in the version for 6–18-year-olds. Examples of items from the first subset are: “Too fearful or anxious,” “Self-conscious or easily embarrassed,” “Shy or timid,” “Unhappy, sad, or depressed,” “Withdrawn, doesn’t get involved with others,” and “Worries.” Examples from the second subset are: “Can’t sit still, restless, or hyperactive,” “Cruel to animals,” “Destroys his own things,” “Destroys things belonging to his family or others,” “Disobedient,” “Doesn’t seem to feel guilty after misbehaving,” “Get in many fights,” “Physically attacks people,” and “Temper tantrums or hot temper.” The CBCL uses a 3-point Likert scale: 0 = *not true*, 1 = *somewhat or sometimes true*, and 2 = *very true or often true*. Internal consistency was high for the externalizing Aggression scale; as estimated by Cronbach’s α, it was .69 in the 1½–5 version and .95 in the 6–18 version. Internal consistencies for the Depression scale were .70 in the 1½–5 version and .86 in the 6–18 version. (Cronbach’s α = .78 to .82 across time points), and moderate for the internalizing scale (Cronbach’s α = .72 to .78).

#### Analysis

We used the Mixed procedure in IBM SPSS 23 to estimate multi-level growth curve models of BMI and CBCL symptoms of aggression and depression.

### Results

#### Demographic characteristics of the sample

In the overweight group and normal-weight group (OWg, NWg), respectively, 93% and 95% of children were firstborn, 96% and 94% of households were intact and all the children were of homogeneous nationality and biological children of their parents. Most families had a middle-class socioeconomic status (SES; 94%) [7]. No female subjects in either the OWg or the NWg reached menarche.

Table 1 presents basic descriptive statistics for individual variables. Whereas BMI was approximately normally distributed in both overweight and normal-weight groups, the distribution of both CBCL scales was markedly positively skewed in the NW group and approximately normal in the OW group.

**Table 1:** Descriptive statistics for study variables across the three phases.

		Age		BMI		AGG		DEP	
Group	Phase	*M*	*SD*	*M*	*SD*	*M*	*SD*	*M*	*SD*
NWg	1	2.22	.26	16.05	1.53	3.86	2.29	1.54	1.41
	2	5.10	.49	16.10	.91	3.61	2.93	1.86	1.91
	3	7.71	.46	16.16	.91	1.04	1.17	.91	1.17
	Total	5.01	2.28	16.10	1.15	2.84	2.58	1.44	1.57
OWg	1	2.40	.53	24.48	2.77	5.44	3.21	3.82	2.55
	2	5.29	.36	26.01	2.38	6.43	2.97	4.54	2.24
	3	7.80	.42	25.93	1.56	10.24	5.14	6.13	2.67
	Total	5.16	2.26	25.47	2.39	7.37	4.40	4.83	2.67

*Note*. N = 90 in each cell. AGG, CBCL Aggression subscale; DEP, CBCL Depression subscale.

#### Developmental trajectories of BMI

**Fig 1:**
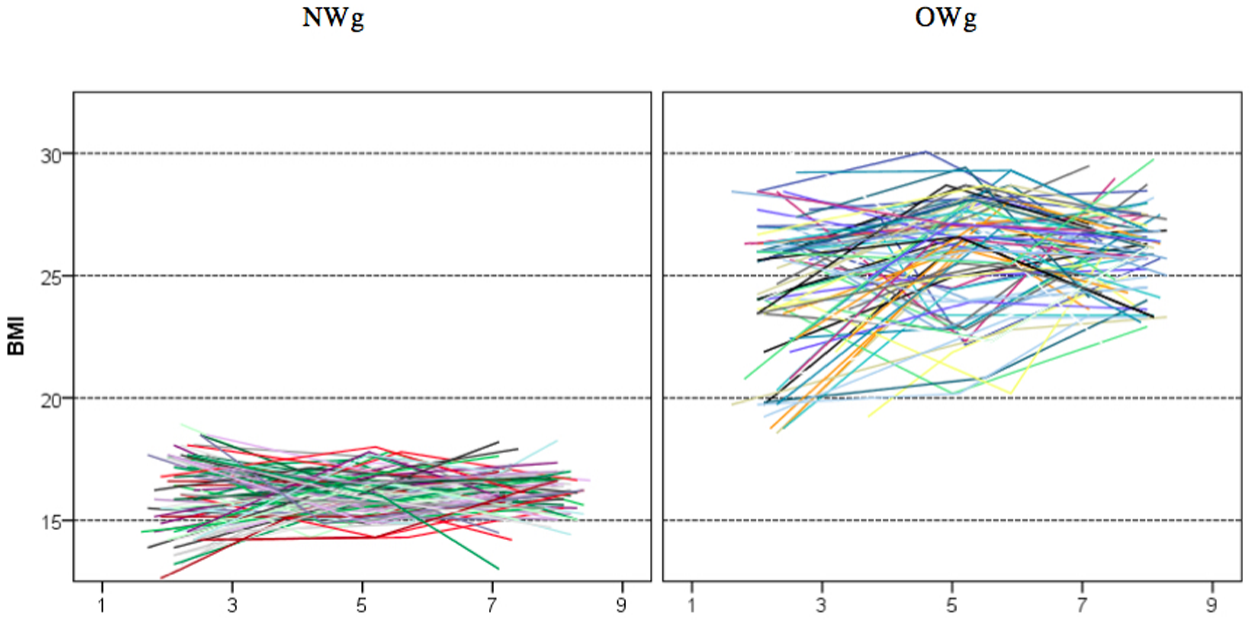
Individual BMI growth curves in the normal-weight and overweight groups.

To describe the developmental trajectories of BMI, we used a growth-curve modelling approach as described by Singer and Willet [59]. In this approach, individual age at the time of measurement is used instead of the rough ordinal phase variable. The small sample size allows us to display all the individual observed growth curves in Fig 1. In the overweight group, the observed individual trajectories started higher than in the normal-weight group and remained higher until the last phase. There is noticeably more variance in the overweight group. Over the course of the study, BMI variance in both groups tended to decrease.

In contrast to clear between-group differences, the differences among individuals within groups appear to be much less systematic. This is also evidenced by the very low correlations (n.s.) between BMIs across phases in the normal-weight group (see Table 2). This raises doubts about whether to model the intercept of BMI developmental trajectories as random, as opposed to fixed. But in the overweight group, the correlations are around .3 (*p* < .05). Since at least in the overweight group the differences in BMIs among children have same stability, we chose to try to model this variability by including random intercept and slope parameters in the model.

**Table 2:** Correlations of BMI across phases. Normal-weight group above diagonal, overweight below.

	**BMI 1**	**BMI 2**	**BMI 3**
**BMI 1**		-.14	-.09
**BMI 2**	.30		-.05
**BMI 3**	.28	.25	

Based on the observed growth trajectories, we specified a linear growth-curve model conditioned on group membership in Model 1 and on group membership and sex in Model 2. Although in an unconditional model it was possible to estimate the variance of both intercepts and slopes, variance of slopes was very small. Further, adding group to the model caused the slope variance to become inestimable. Thus, models presented here include in their stochastic part only residual variance and variance of intercepts; slopes are fixed conditional on group and sex. The results of growth-curve modelling of BMI are reported in Table 3. Both models capture the majority of variance in BMI across phases and individuals. Model 1 captures 89% and Model 2 90% of variance in BMI. The inclusion of sex significantly improved the model in terms of information criteria, but the sex differences are very small.

**Table 3:** Summary of BMI growth-curve models.

Parameter	Model 1		Model 2	
Intercept	15.972	**	16.609	**
Age	.026		-.057	
Group-Overweight	8.055	**	6.206	**
Group-Overweight*Age	.253	**	.483	**
Sex			-1.393	**
Group-Overweight*Sex			3.801	**
Sex*Age			.180	*
Group-Overweight*Sex*Age			-.472	**
Residual VAR	2.72	**	2.60	**
Intercept VAR	.60	**	.51	**
*Information criteria*				
-2LL	2163.8		2132.2	
AIC	2175.8		2152.2	
BIC	2201.5		2195.1	
Parameters	6		10	

*Note*. Group coded 1 for overweight group and 0 for normal-weight group. Sex coded 1 for female and 0 for male. Variance of BMI across individuals and phases = 25.4.

**p < .01,

*p < .05

**Fig 2:**
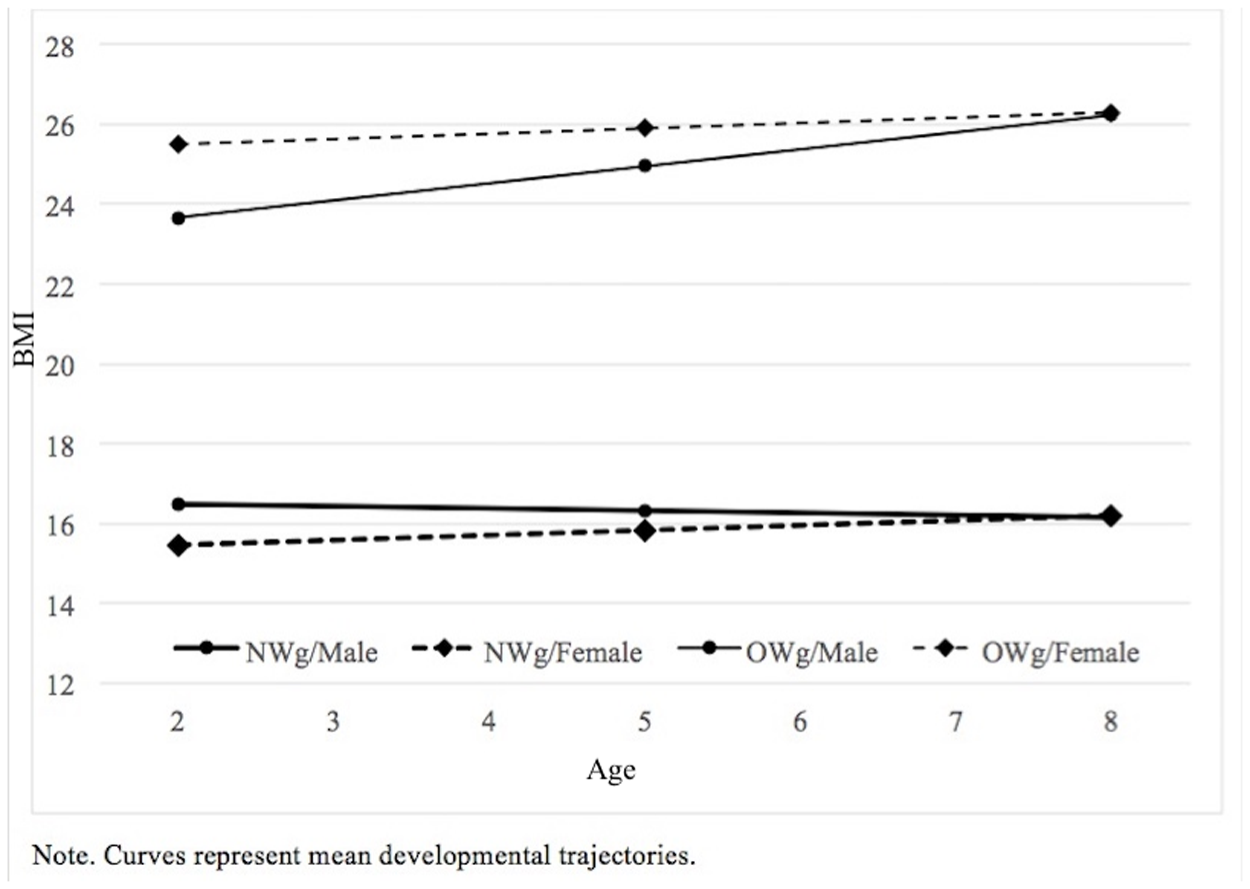
Modelled BMI growth curves from age 2 to 8 by group and sex.

To facilitate interpretation, mean growth curves are plotted in Fig 2. Given that the overweight group was coded 1 and the normal-weight group was coded 0 and age in years was used as the time-variable, the *intercept* in Model 1 is the mean BMI at age 0 in the normal-weight group—the level. The *age* parameter in Model 1 represents the annual BMI increase/decrease in the normal-weight group—the growth rate. The *age* parameter is not significantly different from 0, the predicted linear trajectory in the normal-weight group is flat. Because the *intercept* is modelled as a random parameter, development in the normal-weight group can be described as stable, with the levels of BMI having a normal distribution with mean = 15.97 and SD = .77 (variance in intercepts is estimated at .60). The next two parameters in Table 3 represent the differences in level and growth rate for the overweight group with respect to the normal weight group. The intercept of the mean linear growth trajectory in OWg is 8.06 higher than in NWg (*group-overweight*), i.e. the mean level of the BMI trajectories in OWg is higher than in NWg. The mean growth rate in the OWg is also significantly higher by .25 BMI units per year. Together, the mean intercept in the OWg group is 24.02 (SD = .77) with .28 mean annual increase. Because the growth is not flat, it is important to interpret only mean BMIs within the age range of the study, i.e. from 2 to 8 years.

With sex coded 1 for female and 0 for male in Model 2, the above described parameters represent males’ development in the NWg and OWg groups. The four newly added parameters describe how the mean growth trajectory differs for females in NWg and in OWg. Thus, we can see the mean modelled trajectory level in NWg for females is 1.39 lower than for males (*p* < .01) but in contrast to males’ stable level females’ trajectory is slightly increasing, specifically by .12 annually (*p* < .05). In OWg, females’ modelled mean BMI is 3.80 higher than males’ (*p* < .01). This is, however, compensated for by the fact that the females’ growth rate is significantly slower than males (.13 annually, compared to .42 in males). The variance in intercepts is slightly smaller thanks to the introduction of sex into the model; the growth trajectories are normally distributed with SD = .71, so the majority of the growth trajectories of children within the groups defined by sex and weight status should fit into a range +/-1.4 around the estimated intercept.

#### Developmental trajectories of aggression and depression

**Fig 3:**
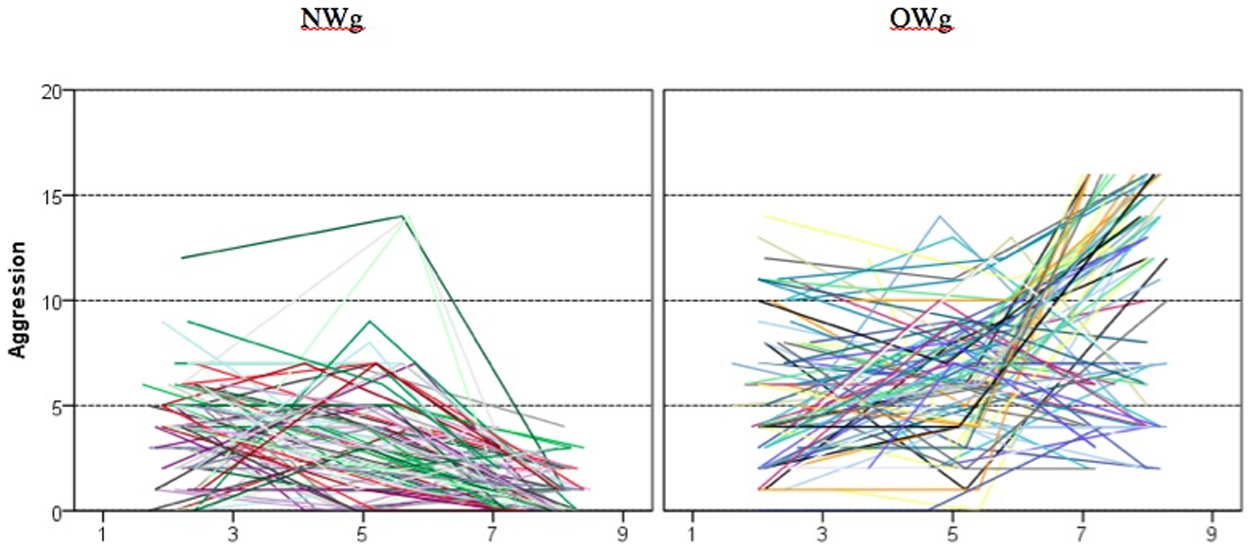
Individual aggression growth curves in the normal weight and overweight groups.

**Fig 4:**
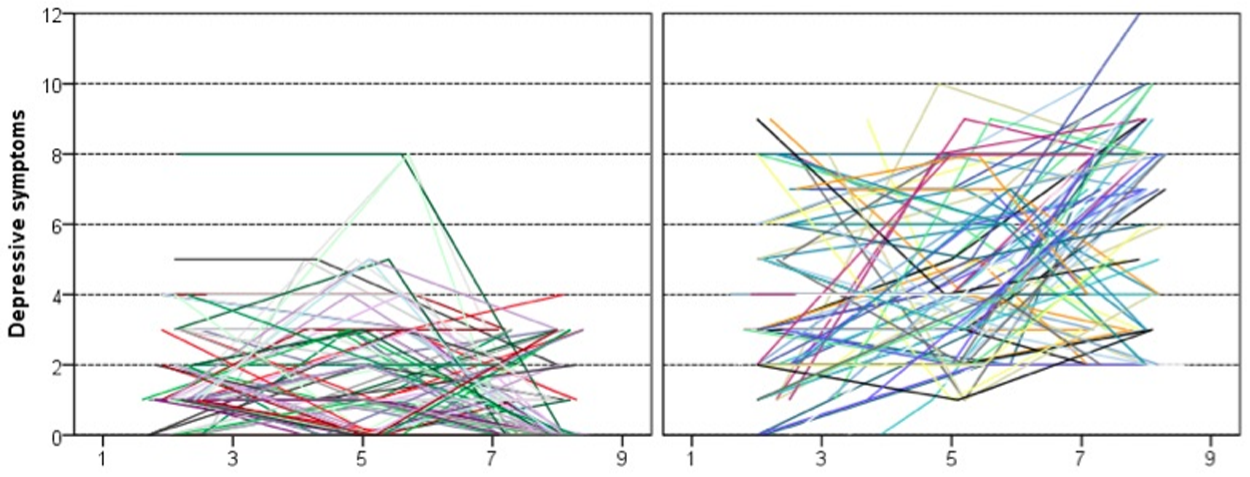
Individual depressive-symptoms growth curves in the normal weight and overweight groups.

To describe the development of behavioral and emotional problems as measured by CBCL over the span of the study, we used the same multilevel approach as for BMI. The individual empirical growth curves are presented in Figs 3 and 4. Aggression trajectories in the normal weight group are more homogenous and generally lower than in the overweight group. Also, the majority shows a decreasing trend, albeit not always a linear one. Finding patterns in the overweight group is more difficult. Yet, it is obvious that there is more aggression reported in the overweight group, and that differences among children in this group increase over time. Although many of the individual trajectories are not linear, having only three measurements allows us to estimate only linear trends. Some of the unexplained variance in the following models is thus due to this nonlinearity of the individual trajectories. Phase-to-phase correlations (Table 4) are very small (and n.s.) in the normal-weight group. In the overweight group, only the correlation between first two measurements (ages 2 and 5) is statistically significant, and it is still small in size. This further documents that within groups children experience quite different development trajectories.

**Table 4:** Correlations of aggression and depressive symptoms scores across phases. Normal weight group above diagonal, overweight below.

	**AGG 1**	**AGG 2**	**AGG 3**		**DEP 1**	**DEP 2**	**DEP 3**
**AGG 1**		.16	-.03	**DEP 1**		.40	-.01
**AGG 2**	.33		-.01	**DEP 2**	.20		-.05
**AGG 3**	.09	-.01		**DEP 3**	-.15	.24	

*Note*. Correlations over .30 are *p* < .01 and those over .21 are *p* < .05.

Similar to BMI models we specified the growth-curve models of aggression and depressive symptoms as linear multi-level models. The models include a linear trend conditioned on group membership in Model 1 and on group membership and sex in Model 2. In the stochastic part of the aggression models, slope variance was fixed; in depression symptom models, it was possible to estimate variance in intercepts and slopes, and their covariance. That means, that unlike in the previous models in this paper, not only the intercept but also the growth rate is modelled as a distribution with a mean at the estimated parameter and some variance. However, in Model 2, none of these variances is significantly different from 0. The summary of the models is reported in Table 5. Both models of aggression captured a substantial proportion of variance in aggression scores across phases and individuals. Model 1 captured 48% and Model 2 57% of variance in aggression. The inclusion of sex significantly improved the model both in terms of interpretability and information criteria. The trajectories for boys and girls in the overweight group substantially differ in the rate of change. To facilitate interpretation mean growth curves are plotted in Fig 4.

**Table 5:** Summary of aggression and depressive symptoms growth-curve models.

	Aggression				Depression			
Parameter	Model 1		Model 2		Model 1		Model 2	
Intercept	4.513	**	4.284	**	1.425	**	1.460	**
Age	-.389	**	-.353	**	-.083		-.075	
Group-Overweight	-1.967	**	-3.630	**	.568		1.952	**
Group-Overweight*Age	1.308	**	1.921	**	.548	**	.166	
Female			.480				-.078	
Group-Overweight*Female			3.860	**			-2.834	**
Female*Age			-.075				-.016	
Group-Overweight*Female*Age			-1.251	**			.782	**
Residual VAR	9.52	**	7.84	**	2.73	**	2.56	**
Intercept VAR	.73		.18		2.11		1.60	
Int-Slope COV					-.34		-.16	
Slope VAR					.07	*	.02	
*Information criteria*								
-2LL	2786.5		2656.7		2203.4		2115.6	
AIC	2798.5		2676.7		2219.4		2139.6	
BIC	2824.2		2719.7		2253.7		2191.1	
*Parameters*	6		10		8		12	

*Note*. Group coded 1 for overweight group and 0 for normal weight group. Sex coded 1 for female and 0 for male. Variance of aggression across individuals and phases = 18.4. Variance of depressive symptoms across individuals and phases = 6.9.

** *p* < .01,

* *p* < .05.

**Fig 5:**
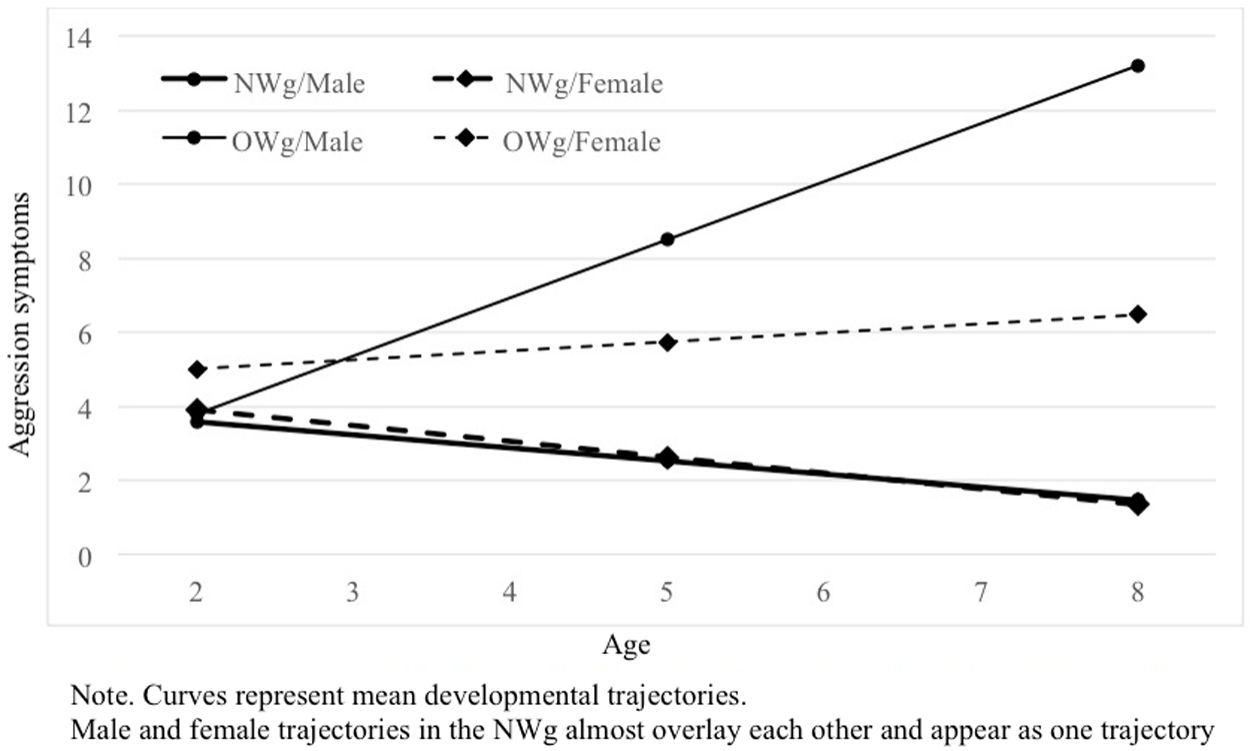
Modelled aggression growth curves from ages 2 to 8 by group and sex.

**Fig 6:**
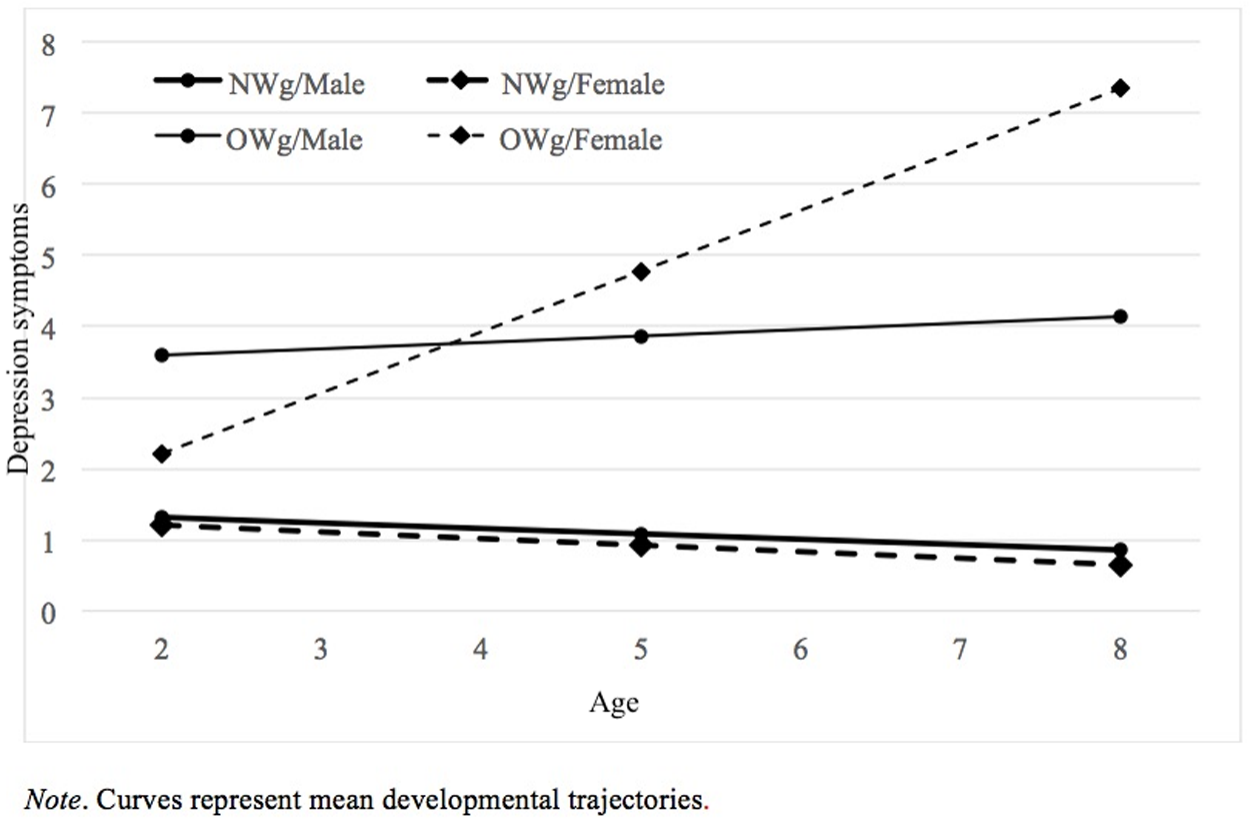
Modelled depression symptoms growth curves from ages 2 to 8 by group and sex.

The models also explained a substantial proportion of variance in depressive symptoms scores across phases and individuals. Model 1 captured 61% and Model 2 63% of variance. Again, the effect of sex was significant in terms of information criteria and interpretability. To facilitate interpretation of aggressive and depressive symptoms, mean growth curves are plotted in Figs 5 and 6.

## Discussion and conclusion

The literature has widely demonstrated that overweight in children can be related to a series of biological, contextual factors [60], but few studies have focused on possible associations between high BMI and mental health in young children. Some studies did suggest that overweight in youths can be associated with psychopathological risk, and in particular with depressive and aggressive symptoms [61]. Nevertheless, most longitudinal studies have focused on BMI and psychological profile trajectories from childhood to adolescence and adulthood in clinical samples, whereas few authors have addressed shorter periods, such as within childhood in community samples [62]. Indeed, studies conducted on community samples recruited in schools can play a crucial role in preventing overweight in children, as the majority of children in a given community attend school [63]. School-based screening programs can measure children’s BMI to identify those at risk for overweight, offering parents information about their offspring’s weight and psychological status to help them take appropriate actions. Previous research has also indicated the necessity of studying associations between children’s BMI and psychopathological symptoms from an early age, separately for males and females, with BMI repeatedly measured throughout childhood. Since several studies demonstrated that parents and children frequently are not aware of youths’ weight status and psychological well-being, school-based screening programs in this field offer the two-fold benefit of objectively evaluating both children’s BMI and possible associated psychological difficulties over time (see, for example, [64]).

In this paper, we aimed to evaluate BMI trajectories from ages 2 to 8 years in two groups of male and female children (overweight and normal weight; OWg/NWg). Moreover, we intended to describe the development over time of children’s aggressive and depressive symptoms during the same time span. In our results, when observing the trajectories of children’s BMI in both groups, without specifically considering sex differences, the weight status of OWg and NWg appeared substantially stable across the three assessment points. Our results indicate that girls show a higher BMI than boys at 2 years old, but boys catch up with them by age 8. So, while females’ BMI was constantly high in the overweight group, males’ BMI was increasing. Our first results are consistent with studies conducted on young children [65], as well as with studies on elementary school children [66,67] suggesting the relative stability of weight status growth during infancy and childhood. However, it is important to note that there are several factors driving differences in growth trajectories [68], such as the effect of genetic variants [69], and early life contextual factors [8]. Moreover, the number of data points, analytic methods, sample characteristics, and other factors also account for differences among studies in growth trajectories [66,67]. Therefore, when examining sex dimorphism, although some studies have found gender differences in diverse samples (e.g., [70]), it is not surprising to observe inconsistent findings of gender differences in weight status trajectories [66]. In summary, considering the diversity of methodologies within human growth research [71], it is important to standardize and reach a consensus about the best model(s) for the study of weight status growth in infancy and childhood [72].

Following Fernandez Castelao and Kröner-Herwig [73], we also verified whether boys and girls in both the OW and NW groups had different trajectories with regards to their aggressive and depressive symptoms. In doing so, we aimed at filling a gap in the literature since a number of studies have demonstrated differences in the phenomenological manifestation of psychopathology in male and female children [74,75] but no study to our knowledge has verified this pattern in the trajectories of overweight children from a very young age to 8 years. With regards to the trajectories of aggressive symptoms, our analyses showed that at T1 (2 years of age) the means of all children were the same. A significant deviation then occurred from the relative stability observed from T1 to T2 in both samples, with divergent directions. In the normal weight group, aggressive symptoms decreased at T3 (8 years of age), with males and females in the NWg remaining on an overlapping trajectory. Conversely, at the same assessment point, in the overweight group these symptoms significantly increased and, specifically for OWg boys, parents reported highest aggressive symptoms. In the case of depressive symptoms, the two groups showed different starting points, with NWg scores significantly lower than OWg scores. Very interestingly, overweight females showed lower depressive scores than OWg males at T1, but they surpassed boys (who remained relatively stable over time) even before T2, showing significantly more maladaptive symptoms at T3. On one hand, the decrease of NWg scores for both aggressive and depressive symptoms is consistent with previous literature indicating that around age 8, children show less problematic emotional/behavioral functioning [76]. On the other hand, while previous longitudinal research on overweight children has suggested that increasing trajectories of maladaptive symptoms are observed in association with linear increasing BMI in childhood, our data shows a higher degree of aggressive and depressive symptoms even with a relatively stable BMI in OWg. Thus, even if children’s BMI does not increase over time, their aggressive and depressive symptoms do. This result should alert both pediatricians and families to be attentive to the psychological well-being of their overweight children, even if their physical conditions do not worsen. Our finding of an early association between aggression and overweight is consistent with previous studies, which demonstrated higher externalizing scores in 2-year-old overweight children. Nevertheless, our work is the first to find this association specifically in overweight males and throughout childhood, from 2 to 8 years. Another study found such behavioral problems in boys, but focused on subjects aged 9–16 [42].

We are not able to ascertain a direction in the association between early aggressive symptoms (at 2 years of age) and high BMI, and therefore, based on our analyses, we cannot affirm what comes first, overweight or aggressive symptoms. Our results only allow us to state that there is a variation in BMI due to contextual factors [77]. Nevertheless, we can speculate that high BMI may result from child management techniques used by parents or caregivers in the rearing of a challenging child (possibly a child with difficult temperament). In an attempt to respond to their offspring’s behavior, parents may engage the child in sedentary activities (e.g. gaming or television watching) or offer food as a reward for behaving, which can negatively alter eating patterns. McEwen [78] has also argued that biological factors influencing brain development could simultaneously be the basis of aggressive behavior and weight gain, and that the developmental paths governing appetite and emotion regulation are interdependent. In our study, we did not observe a difference between male and female children in the shape of increasing trajectories of aggression and depression. This is inconsistent with previous literature, which suggested sex-specific paths in the onset of psychopathological symptoms due to the diverse development of emotional regulation processes in males and females, based on different pathways of brain frontal lobe maturation [79]. It is possible that the age range considered in our study did not allow us to appreciate these differences.

Although we obtained interesting results, our study has some limitations that should be kept in mind. First, we relied upon parental reports of children’s emotional and behavioral functioning, using the CBCL, which is not a diagnostic tool. Nevertheless, several studies have demonstrated that mothers and fathers are reliable sources of information about their offspring’s psychological profiles, and no study has yet shown that parents of overweight children are influenced or biased in describing them [80]. Second, we did not have access to important variables that could be related to or predictive of overweight in children, such as birth weight for gestational age [81]. However, we did have an indication from the parents of whether or not their children were born preterm, ill, or subsequent to a problematic delivery (these conditions were adopted as exclusion criteria). Third, our longitudinal design did not include pubertal age, so we could not verify possible modifications in the trajectories of BMI in males and females, which are likely, as indicated by Dunger, Ahmed, & Ong [82]. At the moment, a further assessment point (T4; 11 years of age) is being performed on the same sample, and the results will be included in a future paper. Fourth, due to the design of the study using groups selected by BMI, the within-group between-subjects variances are artificially low. That prevents the interpretation of individual level differences and correlations. Within-group correlations between BMI and aggression/depression are range-restricted nearly to zero. Only group differences could be interpreted. The across-groups correlations only reflect group differences reflecting the design of the study. While this complex pattern of data is well represented by the growth curve models it may not be so clear to readers with limited experience with growth-curve models. Last, the result showing early high depressive and aggressive symptoms in overweight children should be taken with caution. These symptoms could be linked to other issues (e.g., parental maltreatment) that have not been possible to assess in our study. Thus, further research is needed to clarify the mechanism underpinning the early maladaptive symptoms in OW children.

Notwithstanding the above limitations, this paper has several strengths. First, traditionally, population-based BMI statistical analyses have taken into account whole samples. We chose instead an approach focusing on subgroups of children with an atypical growth. Such an approach is crucial to capturing the heterogeneity in the trajectories of children’s BMIs and psychopathological symptoms [83]. Second, this study examined the developmental trajectories of BMI and emotional-behavioral functioning from early childhood to 8 years of age, which has rarely examined in previous literature on overweight children [84]. Third, we employed widely used tools to measure the study variables and operated a selection of the items in CBCL on the basis of previous literature (as described above), which guaranteed its scores from T1 to T3 to be comparable for both aggressive and depressive symptoms. Finally, our results have important clinical implications. Professionals working in pediatric settings must consider that overweight children need attention both from a biological and psychological standpoint, and be aware that even if BMI does not increase over time in children from 2 to 8 years, their psychopathological symptoms may grow in intensity. Clearly, this issue must be addressed by prevention as well as intervention efforts. Moreover, clinicians must bear in mind that the classical view in which males are more aggressive than females at every age seemingly does not apply to overweight offspring. OW girls, in fact, can be more aggressive than males at 2 years of age (even though higher aggressive symptoms in OW females at T1 could be due to parental over-rating due to cultural stereotypes making them more attuned to girls’ aggressive behavior; [85]). The same logic applies to depressive symptoms, as in our study overweight males at T1 showed a higher degree of depressive symptoms than OW females. From this standpoint, professionals should consider interventions in overweight children’s psychopathological symptoms, tailoring strategies to children’s age and sex alongside their families.

## Abbreviations

BMI: Body Mass Index
CBCL: Child Behavior Check-List
IOTF: International Obesity Task Force
NWg: Normal Weight group
OWg: Underweight group
WHO: World Health Organization
